# Efficacy of Short-Course Antibiotic Therapy for Acute Cholangitis With Positive Blood Cultures: A Retrospective Study

**DOI:** 10.7759/cureus.58883

**Published:** 2024-04-24

**Authors:** Sakue Masuda, Yoshinori Imamura, Chikamasa Ichita, Ryuhei Jinushi, Jun Kubota, Karen Kimura, Makomo Makazu, Ryo Sato, Haruki Uojima, Kazuya Koizumi

**Affiliations:** 1 Gastroenterology Medicine Center, Shonan Kamakura General Hospital, Kamakura, JPN; 2 Division of Medical Oncology/Hematology, University of Fukui Hospital, Fukui, JPN; 3 Department of Gastroenterology, Saitama Medical University international Medical Center, Hidaka, JPN; 4 Department of Gastroenterology, Saitama Medical University International Medical Center, Hidaka, JPN

**Keywords:** duration of antimicrobial therapy, antimicrobial resistance, endoscopic retrograde cholangiopancreatography, cholangitis, antimicrobial stewardship

## Abstract

Background:Short-term treatment of acute cholangitis is sufficient for cure compared with the standard treatment duration. Whether this short-course antimicrobial therapy is effective in patients with acute cholangitis with positive blood cultures has not been fully investigated. This study assessed whether patients with acute cholangitis could achieve successful outcomes with a three-day or shorter antimicrobial treatment period, even with a positive blood culture.

Methods:This single-center retrospective study involved patients with acute cholangitis, defined according to the Tokyo Guidelines 2018 for any cause, who underwent successful biliary drainage and completed a seven-day or shorter antimicrobial treatment. Patients were categorized into six groups based on the duration of antibiotic use (short or standard) after endoscopic retrograde cholangiopancreatography and blood culture findings (positive, negative, or no collection). The primary outcome was the clinical cure rate, defined as no initial presenting symptoms by day 14 after biliary drainage and no recurrence or death by day 30. Secondary outcomes included a three-month recurrence rate and length of hospital stay.

Results:In total, 389 cases were selected, and 27 patients (6.9%) undergoing short-course therapy tested positive for blood culture. The clinical cure rate (n=25, 92.6%) in this group was comparable to that in the other groups. For the three-month recurrence rate (n=1, 3.7%) and median hospital stay (six days), this group's outcomes were either better or similar to those of the other groups.

Conclusions:For cases of successful drainage in acute cholangitis, even with positive blood cultures, short-term antibiotic therapy may be appropriate.

## Introduction

Antibiotic resistance is a growing problem worldwide, and antibiotic-resistant infections are associated with increased morbidity, mortality, and healthcare costs [1,2]. Mitigating antimicrobial drug resistance encompasses strategies such as drug discovery and control of the development of antimicrobial drug-resistant pathogens. Given the protracted research timelines and substantial expenses involved in drug discovery, controlling the development of antimicrobial drug-resistant pathogens is imperative [1,3]. Long-term administration of antimicrobial agents poses a risk for the emergence of multidrug-resistant bacteria [4-7]. Long-term administration of antibiotics in treating acute cholangitis (AC) can lead to an increase in resistant bacteria [8]. Therefore, global recommendations advocate the short-term administration of antimicrobial agents [9,10].

The Tokyo Guidelines 2018 (TG18), the most prominent AC guidelines, recommend 4-7 days as the duration of antibiotic administration after biliary drainage; however, a recent randomized controlled trial compared 4- and 8-day periods and confirmed non-inferiority [11]. Moreover, recent retrospective studies suggested that a 2-3-day course or less of antibiotic therapy following biliary drainage is a reasonable duration for antibiotic administration to treat AC [12-15].

Several retrospective studies have shown that these short-course treatments do not worsen the outcomes, even in patients with AC with positive blood cultures [14,16]. In response, the French Society of Infectious Diseases recommends a short course of antibiotic therapy (within three days), even for cholangitis with bacteremia [9]. However, these studies have some limitations. They did not focus on patients with AC and positive blood cultures; therefore, the characteristics of these patients were not fully presented. In addition, optimal outcomes in AC studies have not been established, and previous studies used various outcomes, making comparisons between studies difficult [12,17]. Furthermore, in previous studies, the short-course treatment group was set at three days or less, using TG18 as a reference. However, the control group included all patients treated for longer than the short-course treatment. In other words, cases with a treatment period longer than the standard treatment period proposed by the TG18 were included [12,17], which is not an appropriate grouping for investigating the non-inferiority of short-course treatment to standard treatment.

Herein, we defined the outcome as a clinical cure based on the Food and Drug Administration guidelines [18] and the study population as patients who received antimicrobials for seven days or less after endoscopic retrograde cholangiopancreatography (ERCP). We hypothesized that patients with AC with positive blood cultures could be treated with short-course antimicrobial therapy if the source of infection was properly drained and analyzed comparative data between the short-course and standard treatment groups.

## Materials and methods

Study design and patient selection

This study is a single-center retrospective investigation, distinct from our other research conducted using the same institutional cholangitis database. Our 2024 publication, titled 'Antimicrobial therapy outcomes in acute cholangitis: Hilar multiple obstructions versus single hilar and common bile duct obstructions [19],' compared the outcomes of patients with different types of obstructions, which was based on the latest insights at the time. While there are methodological similarities due to the shared database and overarching theme of cholangitis, the current study diverges in its focus. Here, we categorize groups based on blood culture results and the durations of antimicrobial treatment, unlike the prior work which was centered around comparing different anatomical sites of obstruction. This methodological shift underscores our aim to delve deeper into the microbial and therapeutic dimensions of acute cholangitis management, highlighting the unique research objectives of the current study.

We included patients who presented with AC at Shonan Kamakura General Hospital between January 2018 and June 2020. AC was defined according to TG18 [20]. Only patients aged ≥18 years who received antibiotic treatment within seven days following successful biliary drainage, as recommended by the TG18, were included. Technical success was defined as the placement of plastic or metallic stents above the bile duct stricture or stones or successful stone extraction [21].

We applied specific exclusion criteria and omitted cases complicated with acute cholecystitis and those at the terminal stage of a malignant tumor. Patients with a preceding severe illness before AC onset, unsuccessful biliary drainage, biliary hemorrhage, a history of intestinal reconstruction methods other than Billroth I, and those in whom mortality status could not be ascertained after 30 days were excluded. Unsuccessful biliary drainage was defined as the inability to achieve technical success in bile duct drainage following one or multiple interventions using ERCP, percutaneous transhepatic biliary drainage, or endoscopic ultrasound. Furthermore, we excluded cases in which antimicrobial therapy after biliary drainage exceeded seven days. Patients with cholangitis recurrence within three months were excluded.

Exposure

The duration of antimicrobial therapy was investigated, with 4-7 days after ERCP as the standard treatment period according to the TG18 guidelines and within three days after ERCP as the short-term treatment period. Blood culture results were categorized as positive, negative, or no collection. The patients were categorized into six groups based on the duration of antibiotic use after ERCP and blood culture findings (Table 1). Special attention was dedicated to comparing groups 1, 2, and 4.

**Table 1:** Group category

Group 1	Short-course duration with positive blood culture
Group 2	Short-course duration with negative blood culture
Group 3	Short-course duration without blood culture collection
Group 4	Standard-course duration with positive blood culture
Group 5	Standard-course duration with negative blood culture
Group 6	Standard-course duration without blood culture collection

Variables and outcomes

The variables included age, comorbidity, cause of AC, severity of AC according to the TG18, National Early Warning Score (NEWS), ERCP findings, antimicrobial therapy, and blood or bile culture findings. For specific factors of particular importance, we listed severity according to the TG18 [20], factors previously reported to exacerbate outcomes in cholangitis (age [22], the Charlson Comorbidity Index (CCI) [23,24], primary disease as a malignant tumor [25,26], multiple hilar biliary strictures [21], and the time from consultation to ERCP [27]), antimicrobial therapy duration [21], and the NEWS within 24 h before the termination of antimicrobial administration [21]. Blood cultures were collected as a principle before antibiotic administration, and bile cultures were collected immediately after ERCP. 

Clinical success of biliary drainage is often defined as a decrease in bilirubin levels of more than 50% from the pretreatment value, measured two weeks after the procedure, based on the Tokyo Criteria 2014 [28]. However, patients with AC are often discharged within a week, and this definition does not allow early determination of the effectiveness of drainage. Therefore, in this study, the clinical success of ERCP was defined as a 50% decrease or normalization of the total bilirubin or alanine aminotransferase level within one week of ERCP.

We employed interpretive standards from the 31st edition of the Clinical and Laboratory Standards Institute for minimum inhibitory concentration or zone diameter testing to identify susceptible or resistant organisms [29]. 

We considered antimicrobial therapy duration an important factor to investigate because while the level of evidence is low, previous reports indicate that the antimicrobial therapy duration can be shortened compared to that recommended in the TG18 [12-14].

To standardize the conditions of patients immediately before discontinuation of antibiotics, we incorporated the NEWS into our analysis. The United Kingdom’s National Early Warning Score Development and Implementation Group developed the NEWS in 2012 to assess deteriorating conditions in hospitalized patients and predict inpatient death or intensive care unit (ICU) admission [30]. The NEWS measures physiological parameters (systolic blood pressure, pulse rate, respiratory rate, temperature, and oxygen saturation), consciousness level, and oxygen supplementation, all of which are simple and easily accessible [30,31]. The reports placed the low-risk group for NEWS at ≤4 points. The NEWS is widespread in many countries because of its greater ability to identify patients at risk for the composite outcome of cardiac arrest, unexpected ICU admission, and death within 24 h than other early warning scores. 

The Deyo modification of the CCI was used to define the severity of the comorbid conditions [31]. A CCI score of ≥4 has been reportedly associated with AC outcomes [23,24,33]. Chronic heart failure was defined as a diagnosis by a cardiologist or general internist or previous hospitalization for heart failure treatment. Chronic kidney disease was defined according to the Kidney Disease Improving Global Outcomes [34]. 

The primary outcome was clinical cure, and the secondary outcomes were the three-month recurrence rate and length of hospital stay. Clinical cure was defined as no initial presenting symptoms by day 14 post-biliary drainage and no recurrence or death by day 30 [11]. Recurrence was defined as the initiation of a new antibiotic therapy for recurrent cholangitis, subsequent infection in the hepatic-pancreatic-biliary region, or any other subsequent infection possibly related to the initial episode of cholangitis [13,16,35].

Statistical analysis

Six groups were classified based on the duration of antibiotic use after ERCP and blood culture results were compared using univariate analysis. 

Continuous and categorical variables were reported as medians and interquartile ranges and numbers and percentages, respectively. Continuous and categorical variables were compared using the Mann-Whitney U and chi-square tests, respectively. Risk differences with 95% confidence intervals (CIs) were calculated for binary outcomes, and a two-sided significance level for all tests was set at p<0.05. All analyses were performed using EZR version 1.55 [35], a package for R statistical software (https://www.r-project.org/), which is a modified version of the R commander designed to add statistical functions frequently used in biostatistics.

## Results

Patient characteristics

**Figure 1:**
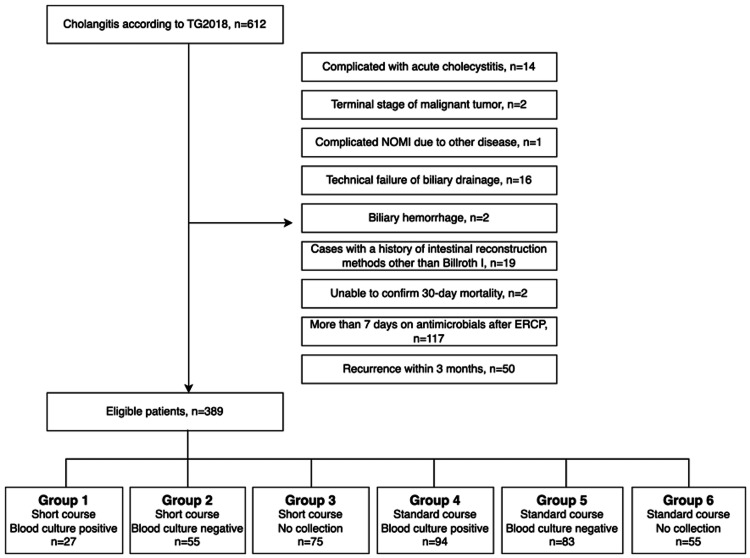
Flowchart presenting the participants' inclusion and exclusion criteria NOMI, non-occlusive mesenteric ischemia

A total of 389 patients with AC were included in this study (Figure 1). Table 2 summarizes the patient characteristics. The characteristics of patients in group 1 included a higher prevalence of stone-related causes of AC, a higher number of severe cases and facility residents, and a significant proportion of cases with high NEWS scores before the completion of antibiotic treatment. However, there were fewer cases with a high CCI. Compared to group 1, group 2 had a higher proportion of cases with malignant biliary strictures and high CCI but fewer cases with severe grades, nursing home residents, and high NEWS scores before the completion of antibiotic treatment. Compared to group 1, group 4 had a higher number of severe cases but fewer cases with high NEWS scores before the completion of antibiotic treatment.

**Table 2:** Patient characteristics Data for binary variables are presented as n (%), while continuous variables are represented as Median [IQR]. Additionally, a p-value of less than 0.05 (p<0.05) was considered statistically significant. AC, acute cholangitis; CCI, Charlson Comorbidity Index; CHF, chronic heart failure; CKD, chronic kidney disease; DM, diabetes mellitus; IQR, interquartile range; LC, liver cirrhosis; NEWS, National Early Warning Score

-	Group 1	Group 2	Group 3	Group 4	Group 5	Group 6	p-value
Variables, n	27	55	75	94	83	55	-
Age, median [IQR]	80.0 [76.50, 86.00]	78.0 [67.00, 85.00]	75.0 [65.50, 82.00]	83.0 [76.00, 89.00]	84.0 [76.50, 89.50]	79.0 [70.50, 85.00]	<0.001
Male, n (%)	15 (55.6)	28 (51.9)	40 (53.3)	46 (48.9)	35 (42.2)	27 (49.1)	0.739
Cause of cholangitis, n (%)	-	-	-	-	-	-	<0.001
Bile duct stone	23 (85.2)	42 (76.4)	37 (49.3)	79 (84.0)	67 (80.7)	28 (50.9)	-
Malignant stricture	4 (14.8)	13 (23.6)	36 (48.0)	15 (16.0)	15 (18.1)	27 (49.1)	-
Benign stricture & others	0 (0.0)	0 (0.0)	2 (2.7)	0 (0.0)	1 (1.2)	0 (0.0)	-
Severity of AC, n (%)	-	-	-	-	-	-	<0.001
Mild	12 (44.4)	28 (50.9)	47 (62.7)	33 (35.1)	32 (38.6)	31 (56.4)	-
Moderate	11 (40.7)	25 (45.5)	26 (34.7)	38 (40.4)	47 (56.6)	21 (38.2)	-
Severe	4 (14.8)	2 (3.6)	2 (2.7)	23 (24.5)	4 (4.8)	3 (5.5)	-
Underlying medical conditions, n (%)	-	-	-	-	-	-	-
CCI (median [IQR])	1.00 [0.00, 1.00]	1.00 [0.00, 2.00]	1.00 [0.00, 2.00]	1.00 [0.00, 1.00]	1.00 [0.00, 1.00]	0.00 [0.00, 1.50]	0.297
CCI≥4	0 (0.0)	5 (9.1)	8 (10.7)	1 (1.1)	5 (6.0)	4 (7.3)	0.078
CKD	4 (14.8)	4 (7.3)	4 (5.3)	7 (7.4)	8 (9.6)	5 (9.1)	0.731
CHF	2 (7.4)	5 (9.1)	2 (2.7)	11 (11.7)	9 (10.8)	7 (12.7)	0.337
LC	1 (3.7)	1 (1.8)	6 (8.0)	4 (4.3)	3 (3.6)	2 (3.6)	0.633
DM	3 (11.1)	12 (21.8)	13 (17.3)	18 (19.1)	19 (22.9)	8 (14.5)	0.692
Hemodialysis	0 (0.0)	3 (5.5)	1 (1.3)	0 (0.0)	0 (0.0)	2 (3.6)	0.066
Constant placement of urinary catheter	0 (0.0)	0 (0.0)	1 (1.3)	0 (0.0)	0 (0.0)	0 (0.0)	0.521
Aspiration pneumonia	0 (0.0)	1 (1.8)	0 (0.0)	4 (4.3)	2 (2.4)	1 (1.8)	0.483
Residence nursing home	8 (29.6)	9 (16.4)	6 (8.0)	26 (27.7)	27 (32.5)	3 (5.5)	<0.001
Immunosuppressant user	0 (0.0)	0 (0.0)	2 (2.7)	4 (4.3)	1 (1.2)	0 (0.0)	0.287
Highest NEWS within 24 h before the termination of antimicrobial administration (median [IQR])	1.0 [1.0, 3.0]	1.0 [0.0, 1.5]	1.0 [0.0, 2.0]	1.0 [0.0, 2.0]	1.0 [0.0, 2.0]	1.0 [0.0, 2.0]	0.115
Highest NEWS ≥5 within 24 h before the termination of antimicrobial administration, n (%)	3 (12.0)	0 ( 0.0)	2 ( 2.7)	1 ( 1.1)	0 ( 0.0)	1 ( 1.8)	0.003

ERCP findings

Table 3 outlines the ERCP findings. In group 1, there were more cases with a longer interval from the initiation of treatment for AC to ERCP. Multiple biliary strictures in the hepatic hilum, which are prone to drainage issues, were less common in group 1. Compared to group 1, group 2 had a higher number of cases with a shorter interval from the initiation of treatment for AC to ERCP and a higher proportion of cases with multiple biliary strictures in the hepatic hilum. Compared to group 1, group 4 had a higher number of cases with a shorter interval from the initiation of treatment for AC to ERCP and a higher number of cases with stent placement. There were no significant differences in clinical unsuccess of ERCP and ERCP-specific complications between the groups.

**Table 3:** ERCP findings Data for binary variables are presented as n (%). Additionally, a p-value of less than 0.05 (p<0.05) was considered statistically significant. IQR, interquartile range; ERCP, endoscopic retrograde cholangiopancreatography; ENBD, endoscopic nasobiliary drainage.

-	Group 1	Group 2	Group 3	Group 4	Group 5	Group 6	p-value
Variables, n	27	55	75	94	83	55	-
Time from consultation to endoscopic retrograde cholangiopancreatography, n (%)	-	-	-	-	-	-	0.001
≤24	15 (55.6)	35 (63.6)	49 (65.3)	75 (79.8)	72 (86.7)	42 (76.4)	-
24–48	4 (14.8)	13 (23.6)	13 (17.3)	15 (16.0)	8 (9.6)	9 (16.4)	-
>48	8 (29.6)	7 (12.7)	13 (17.3)	4 (4.3)	3 (3.6)	4 (7.3)	-
ERCP drainage procedure, n (%)	-	-	-	-	-	-	<0.001
Stone extraction	20 (76.9)	39 (70.9)	30 (40.0)	47 (50.0)	53 (63.9)	20 (36.4)	-
Plastic stent	4 (15.4)	10 (18.2)	27 (36.0)	38 (40.4)	19 (22.9)	14 (25.5)	-
Self-expandable metallic stent	2 (7.7)	4 (7.3)	15 (20.0)	4 (4.3)	4 (4.8)	14 (25.5)	-
ENBD	0 (0.0)	0 (0.0)	1 (1.3)	3 (3.2)	1 (1.2)	2 (3.6)	-
Others	0 (0.0)	1 (1.8)	1 (1.3)	0 (0.0)	0 (0.0)	0 (0.0)	-
Stone extraction and stent placement/ENBD/others	0 (0.0)	1 (1.8)	0 (0.0)	1 (1.1)	3 (3.6)	1 (1.8)	-
Technical unsuccess of ERCP	0 (0.0)	0 (0.0)	1 (1.3)	1 (1.1)	3 (3.6)	4 (7.3)	-
Clinical unsuccess of ERCP	0 (0.0)	3 (5.5)	5 (6.7)	3 (3.2)	1 (1.2)	2 (3.6)	0.26
Bismuth classification, n (%)	-	-	-	-	-	-	<0.001
I	4 (14.8)	6 (10.9)	28 (37.3)	11 (11.7)	12 (14.5)	19 (34.5)	-
II or higher	0 (0.0)	7 (12.7)	10 (13.3)	4 (4.3)	4 (4.8)	8 (14.5)	-
Bile duct stone/benign stricture/Others	23 (85.2)	42 (76.4)	37 (49.3)	79 (84.0)	67 (80.7)	28 (50.9)	-
Complications, n (%)	-	-	-	-	-	-	-
Pancreatitis	0 (0.0)	0 (0.0)	0 (0.0)	3 (3.2)	2 (2.4)	1 (1.8)	0.469
Bleeding	1 (3.7)	1 (1.8)	2 (2.7)	1 (1.1)	3 (3.6)	1 (1.8)	0.894
Perforation	0 (0.0)	0 (0.0)	0 (0.0)	0 (0.0)	0 (0.0)	0 (0.0)	NA
Others	0 (0.0)	0 (0.0)	0 (0.0)	0 (0.0)	0 (0.0)	3 (5.5)	0.003
No complications	24 (96.0)	53 (96.4)	73 (97.3)	89 (95.7)	78 (94.0)	50 (90.9)	0.64

Microbial culture findings

The microbial culture results are summarized in Table 4. Blood culture results were comparable between the groups. In bile cultures, multiple bacteria were commonly detected, and all were counted. Escherichia coli and Enterococcus spp. were more prevalent in groups 1, 4, and 5, whereas Klebsiella spp. were more common in groups 4 and 5.

**Table 4:** Culture results Data for binary variables are presented as n (%).

-	Group 1	Group 2	Group 3	Group 4	Group 5	Group 6
Variables, n	27	55	75	94	83	55
Blood culture, n (%)	-	-	-	-	-	-
Escherichia coli	12 (44.4)	NA	NA	52 (55.3)	NA	NA
Klebsiella sp.	6 (22.2)	NA	NA	21 (22.3)	NA	NA
Enterococcus sp.	4 (14.8)	NA	NA	7 (7.4)	NA	NA
Enterobacter sp.	3 (11.1)	NA	NA	5 (5.3)	NA	NA
Citrobacter sp.	2 (7.4)	NA	NA	2 (2.1)	NA	NA
Streptococcus sp.	0 (0.0)	NA	NA	6 (6.4)	NA	NA
Pseudomonas sp.	0 (0.0)	NA	NA	1 (1.1)	NA	NA
Anaerobes	3 (11.1)	NA	NA	10 (10.6)	NA	NA
Others	3 (11.1)	NA	NA	9 (9.6)	NA	NA
Bile culture, n (%)	-	-	-	-	-	-
No collection	1 (3.7)	3 (5.5)	6 (8.0)	2 (2.1)	1 (1.2)	3 (5.5)
Negative	2 (7.4)	19 (34.5)	24 (32.0)	3 (3.2)	6 (7.2)	8 (14.5)
Positive rate	24 (88.9)	33 (60.0)	45 (60.0)	89 (94.7)	76 (91.6)	44 (80.0)
Escherichia coli	13 (48.1)	7 (12.7)	13 (17.3)	50 (53.2)	36 (43.4)	15 (27.3)
Klebsiella sp.	4 (14.8)	8 (14.5)	19 (25.3)	34 (36.2)	23 (27.7)	9 (16.4)
Enterococcus sp.	13 (48.1)	14 (25.5)	15 (20.0)	37 (39.4)	36 (43.4)	15 (27.3)
Enterobacter sp.	6 (22.2)	4 (7.3)	6 (8.0)	9 (9.6)	11 (13.3)	8 (14.5)
Citrobacter sp.	2 (7.4)	6 (10.9)	4 (5.3)	7 (7.4)	4 (4.8)	3 (5.5)
Streptococcus sp.	1 (3.7)	3 (5.5)	7 (9.3)	7 (7.4)	8 (9.6)	5 (9.1)
Pseudomonas sp.	1 (3.7)	2 (3.6)	3 (4.0)	3 (3.2)	4 (4.8)	3 (5.5)
Anaerobes	2 (7.4)	2 (3.6)	7 (9.3)	6 (6.4)	9 (10.8)	2 (3.6)
Others	2 (7.4)	2 (3.6)	3 (4.0)	7 (7.4)	7 (8.4)	9 (16.4)

Antimicrobial therapy 

Tables 5, 6 summarize the antimicrobial therapies used in the present study. The most commonly used antibiotic was cefmetazole, followed by piperacillin/tazobactam and ampicillin/sulbactam. The broad-spectrum antibiotic piperacillin/tazobactam was commonly used in groups 1 and 4. The duration of antibiotic administration after ERCP was shorter in groups 1-3 than in groups 4-6. 

**Table 5:** Breakdown of the antibiotics used in this study Data for binary variables are presented as n (%). IQR, interquartile range

-	Group 1	Group 2	Group 3	Group 4	Group 5	Group 6
Variables, n	27	55	75	94	83	55
Cefmetazole, n (%)	16 (59.3)	38 (69.1)	57 (76.0)	46 (48.9)	50 (60.2)	41 (74.5)
Piperacillin/tazobactam	6 (22.2)	4 (7.3)	6 (8.0)	22 (23.4)	11 (13.3)	3 (5.5)
Ampicillin/sulbactam	4 (14.8)	8 (14.5)	2 (2.7)	19 (20.2)	16 (19.3)	2 (3.6)
Ceftriaxone	0 (0.0)	2 (3.6)	7 (9.3)	3 (3.2)	1 (1.2)	6 (10.9)
Meropenem	1 (3.7)	1 (1.8)	1 (1.3)	2 (2.1)	2 (2.4)	0 (0.0)
Ciprofloxacin	0 (0.0)	1 (1.8)	0 (0.0)	2 (2.1)	1 (1.2)	3 (5.5)
Vancomycin	0 (0.0)	0 (0.0)	0 (0.0)	0 (0.0)	0 (0.0)	0 (0.0)
Others	0 (0.0)	1 (1.8)	2 (2.7)	0 (0.0)	1 (1.2)	0 (0.0)

**Table 6:** Antimicrobial therapy Data for binary variables are presented as n (%), while continuous variables are represented as Median [IQR]. Additionally, a p-value of less than 0.05 (p<0.05) was considered statistically significant.

-	Group 1	Group 2	Group 3	Group 4	Group 5	Group 6	p-value
Variables, n	27	55	75	94	83	55	-
Duration of antimicrobial therapy after biliary drainage, median days [IQR]	2.0 [2.0, 3.0]	2.0 [1.5, 3.0]	2.0 [1.0, 3.0]	5.0 [4.0, 6.0]	5.0 [4.0, 6.0]	5.0 [4.0, 6.0]	<0.001
Antimicrobial susceptibility for blood culture, n (%)	-	-	-	-	-	-	0.53
Resistant bacteria	5 (18.5)	NA	NA	12 (12.8)	NA	NA	-
Susceptible bacteria	21 (77.8)	NA	NA	82 (87.2)	NA	NA	-
Unknown	1 (3.7)	NA	NA	0 (0.0)	NA	NA	-
Antimicrobial susceptibility for blood and bile cultures, n (%)	-	-	-	-	-	-	<0.001
Resistant bacteria	16 (59.3)	23 (41.8)	18 (24.0)	36 (38.3)	34 (41.0)	25 (45.5)	-
Susceptible bacteria	11 (40.7)	10 (18.2)	27 (36.0)	58 (61.7)	42 (50.6)	19 (34.5)	-
Unknown	0 (0.0)	22 (40.0)	30 (40.0)	0 (0.0)	7 (8.4)	11 (20.0)	-

The susceptibility of the bacteria identified in the blood cultures to the administered antibiotics was comparable among the groups. When considering all bacteria identified in the blood and bile cultures as causative organisms, group 1 had a higher proportion of causative organisms with resistance to the administered antibiotics.

Clinical outcomes

Table 7 presents the clinical outcomes of patients. In group 1, the clinical outcomes were as follows: the rate of clinical cure was 92.6% (n=25), the recurrence rate was 3.7% (n=1), and the median hospital stay was six days. There was no significant difference in the primary outcome, the clinical cure rate, between the groups. Regarding secondary outcomes, there was almost no difference in the three-month recurrence rates between groups 1, 2, and 4, while the duration of hospital stay was shorter in groups 1 and 2 than in group 4.

**Table 7:** Clinical outcomes Data for binary variables are presented as n (%), while continuous variables are represented as Median [IQR]. Additionally, a p-value of less than 0.05 (p<0.05) was considered statistically significant. IQR, interquartile range

-	Group 1	Group 2	Group 3	Group 4	Group 5	Group 6	p.value
Variables, n	27	55	75	94	83	55	-
Clinical cure, n (%)	25 (92.6)	55 (100.0)	70 (93.3)	89 (94.7)	81 (97.6)	52 (94.5)	0.395
Three-month recurrence, n (%)	-	-	-	-	-	-	0.017
Recurrence	1 (3.7)	3 (5.5)	13 (17.3)	6 (6.4)	3 (3.6)	5 (9.1)	-
No recurrence	23 (85.2)	50 (90.9)	58 (77.3)	81 (86.2)	80 (96.4)	48 (87.3)	-
Unknown	3 (11.1)	2 (3.6)	4 (5.3)	7 (7.4)	0 (0.0)	2 (3.6)	-
Length of hospital stay, median [IQR]	6.0 [5.0, 7.0]	6.0 [5.0, 8.5]	6.0 [4.0, 8.0]	8.0 [6.0, 10.0]	7.0 [6.0, 8.5]	7.0 [6.0, 8.0]	<0.001

Cases without clinical cure were a minority, totaling 17 cases (4.3%); their characteristics are presented in Supplementary Table 8. 

## Discussion

We compared the outcomes between patients categorized into six groups based on the duration of antibiotic treatment after ERCP and blood culture results. We found no significant difference in clinical cure in the univariate analysis. Although group 1 had more cases of cholelithiasis and fewer hilar strictures, there was no noticeable clinical deterioration in terms of the severity of cholangitis, patient background as indicated by the CCI, residence in a nursing home, general condition as indicated by the NEWS, time from consultation to ERCP, or the presence or absence of antibiotic resistance.

 Shortening the duration of antibiotic treatment is reportedly as effective as standard treatment in cases of acute cholangitis with positive blood cultures [14,16]. Once the source of infection is controlled by biliary drainage, bacteremia is likely to resolve, potentially obviating the need for further antibiotic therapy [37]. However, these studies did not focus on cases with blood culture findings and did not examine all the factors previously reported to affect the outcome of AC. Furthermore, they used a control group whose treatment duration differed from the TG18-recommended standard treatment duration of 4-7 days, as they also included cases treated for >8 days. We addressed these issues by limiting our study participants to cases with an antimicrobial treatment duration within seven days after biliary drainage, dividing the groups based on blood culture findings and investigating factors previously reported to affect the outcome of AC. Our results indicate that antimicrobial therapy may be safely terminated in patients with AC with positive blood cultures after appropriate biliary drainage. Although it is challenging to fully align our definition of outcome with that of previous reports, in our study, group 1 achieved a clinical cure rate of 92.6% (n=25), which is not inferior when compared to the results of previous reports on AC [12-14]. Three-month recurrence was more frequent in groups 3 and 6 and less common in blood culture-positive groups 1 and 4. This is likely because groups 3 and 6 had more cases of perihilar cholangiocarcinoma and are prone to malignant biliary strictures and poor drainage [21].

Treatment for seven days or less, instead of the traditional two weeks, is sufficient for various infections, even with positive blood cultures [38-40]. This seems to be the case even in immunodeficient states such as febrile neutropenia [38]. However, there are reports of worse outcomes in patients with non-community-acquired bacteremia, whose pathogens tend to show increased antibiotic resistance [40]. Therefore, further research is needed on patients with AC with positive blood cultures who carry a risk of resistant bacteria. In this study, antibiotic resistance in causative organisms was more common in group 1; however, the clinical cure rate in group 1 was favorable. This is likely due to the effectiveness of appropriate biliary drainage in the clinical cure of AC [14,41,42].

These results should be interpreted with caution, considering some limitations. First, this was a single-center retrospective study, and each physician in charge determined the duration of antimicrobial therapy and blood culture sampling. Therefore, severe cases according to TG18 were disproportionately found in groups 1 and 4. Furthermore, the number of cases in group 1 and the number of events without clinical cure were small in this study. Additionally, as this study was descriptive in nature, it did not adjust for confounding factors. Therefore, validation through prospective studies is necessary, and appropriate plans are currently being developed. Second, we could not assess long-course complications, particularly infective endocarditis, which is a concern for gram-positive cocci. It is unclear whether prolonged antibiotic therapy for gram-positive cocci can reduce the risk of infective endocarditis, necessitating further research. The strength of this study is that it set appropriate outcomes for infectious diseases, set up a proper control group following the current guidelines for cholangitis, and focused on blood culture.

## Conclusions

In conclusion, our findings suggest that short-term antibiotic therapy may be deemed appropriate for AC cases with successful drainage, even when blood cultures are positive. Nevertheless, given the nature of this descriptive, single-center, retrospective study, further validation through prospective research is necessary. Plans to undertake such research are actively being formulated.

## Appendices

**Table 8:** Characteristics of patients who did not show clinical improvement AC, acute cholangitis

	No clinical improvement	Clinical improvement
	n=17	n=372
Age, median [IQR]	80.2 (10.2)	78.6 (12.0)
Malignant stricture, n (%)	11 (64.7)	99 (26.6)
Bismuth classification, n (%)		
I	7 (41.2)	73 (19.6)
II or higher	4 (23.5)	29 (7.8)
Bile duct stone/benign stricture/Others	6 (35.3)	270 (72.6)
Blood culture, n (%)		
Negative	2 (11.8)	136 (36.6)
Positive	7 (41.2)	114 (30.6)
No collection	8 (47.1)	122 (32.8)
Severity of AC, n (%)		
Mild	7 (41.2)	176 (47.3)
Moderate	8 (47.1)	160 (43.0)
Severe	2 (11.8)	36 (9.7)
CCI≥4, n (%)	2 (11.8)	21 (5.6)
Technical unsuccess of ERCP, n (%)	0 (0.0)	3 (0.8)
Time from consultation to endoscopic retrograde cholangiopancreatography, median [IQR]		
≤24	14 (82.4)	274 (73.7)
24–48	2 (11.8)	60 (16.1)
>48	1 (5.9)	38 (10.2)
Organisms from bile culture, n (%)		
Escherichia coli	5 (29.4)	129 (34.7)
Klebsiella sp.	0 (0.0)	44 (11.8)
Enterococcus sp.	5 (29.4)	125 (33.6)
Enterobacter sp.	6 (35.3)	91 (24.5)
Antimicrobial susceptibility for blood and bile cultures, n (%)		
Resistant bacteria	7 (41.2)	145 (39.0)
Susceptible bacteria	5 (29.4)	162 (43.5)
Unknown	5 (29.4)	65 (17.5)
Antibiotics, n (%)		
Cefmetazole	10 (58.8)	238 (64.0)
Piperacillin/tazobactam	4 (23.5)	48 (12.9)
Ampicillin/sulbactam	2 (11.8)	49 (13.2)
Ceftriaxone	0 (0.0)	19 (5.1)
Short-course duration of antibiotics, n (%)	7 (41.2)	150 (40.3)
Highest NEWS of ≥5 within 24 h before the termination of antimicrobial administration, n (%)	1 (7.1)	6 (1.6)
